# Combined EP300 and CREBBP Expression Levels Regulate Human Trophoblast Differentiation

**DOI:** 10.3390/ijms27177623

**Published:** 2026-08-25

**Authors:** Fangxu Lin, Remco Keijser, Gijs Afink

**Affiliations:** Reproductive Biology Laboratory, Amsterdam Reproduction and Development, Amsterdam UMC, University of Amsterdam, 1105 AZ Amsterdam, The Netherlands; l.lin@amsterdamumc.nl (F.L.); r.keijser@amsterdamumc.nl (R.K.)

**Keywords:** E1A-binding protein p300, CREB-binding protein, extravillous trophoblasts, trophoblast differentiation, placenta

## Abstract

Extravillous trophoblast (EVT) differentiation is essential for placental development and successful pregnancy, yet the molecular mechanisms regulating this process remain incompletely understood. CREB-binding protein (CREBBP) and E1A-binding protein p300 (EP300) are closely related lysine acetyltransferases that function as transcriptional co-activators. Previous studies reported that EP300 depletion impairs EVT differentiation whereas CREBBP depletion has little or no effect. We found that *CREBBP* mRNA expression was approximately one-third of *EP300* expression in trophoblast stem cells (TSCs) and differentiated trophoblast lineages. To investigate whether differential CREBBP and EP300 expression explains this asymmetry in EVT differentiation initiation, we combined siRNA-mediated knockdown, CRISPR-generated *CREBBP*-deficient TSCs, pharmacological inhibition, and lentiviral *Crebbp* overexpression during EVT differentiation. While *CREBBP* depletion alone produced minimal phenotypic effects, *CREBBP*-deficient cells displayed increased sensitivity to the CREBBP/EP300 inhibitor A-485, with inhibitory effects appearing at lower A-485 concentrations in cells with reduced CREBBP level. Moreover, increased CREBBP expression partially rescued the EP300-depletion phenotype, including EVT-like morphology and selected differentiation marker expression. Together, these findings demonstrate that CREBBP contributes to EVT differentiation, but its role is obscured by its lower endogenous expression. We propose that the combined contributions of CREBBP and EP300, rather than EP300-specific activity alone, influence human trophoblast differentiation efficiency.

## 1. Introduction

The placenta is a dynamic regulatory organ essential for pregnancy, supporting fetal development through nutrient exchange, endocrine signaling, and immune adaptation [1,2,3,4]. Its function depends on the differentiation of cytotrophoblast (CTB) progenitors into two primary lineages [5,6,7]: extravillous trophoblasts (EVTs), which remodel maternal spiral arteries to ensure adequate maternal blood supply to the placenta [5,8], and syncytiotrophoblasts (STBs), which form a multinucleated barrier for hormone secretion and transport [5,9,10]. Defects in trophoblast differentiation are directly linked to pregnancy complications including preeclampsia, fetal growth restriction, and pregnancy loss [6,11,12]. Because trophoblast lineage specification relies on coordinated activation of lineage-specific transcriptional programs, understanding the molecular mechanisms that govern these processes is essential for defining the basis of normal placental development and disease [5,6,7,11,12].

CREB-binding protein (CREBBP) and E1A-binding protein p300 (EP300) are closely related lysine acetyltransferases (KATs) that function as transcriptional co-activators, chromatin regulators and modulators of histone and non-histone protein function in eukaryotic gene regulation [13,14]. At the molecular level, both proteins catalyze lysine acetylation on histone and non-histone substrates, thereby modulating chromatin accessibility, enhancer activity, and transcription factor function [15]. In addition, they serve as molecular scaffolds that facilitate interactions between transcription factors and the basal transcriptional machinery. Through these mechanisms, EP300 and CREBBP play central roles in coordinating gene expression programs that underlie diverse cellular processes, including proliferation, differentiation, and cell fate specification [13,14,16].

EP300 has recently been identified as an important regulator of EVT differentiation in human trophoblast stem cells [17]. Although EP300 and its closely related paralog CREBBP share extensive structural similarity and possess highly conserved acetyltransferase domains, accumulating evidence suggests that they are not universally interchangeable [18,19,20,21,22]. In human trophoblast stem cells, depletion of EP300 impairs EVT formation, whereas depletion of CREBBP produces minimal or no detectable phenotype [17]. These findings suggest that the functional relationship between CREBBP and EP300 in trophoblast differentiation is not fully redundant, but the extent and mechanistic basis of this asymmetry remain unclear.

Together, these findings raise the question of whether CREBBP and EP300 may play distinct or partially overlapping roles during trophoblast differentiation. One possibility is that EP300 has a non-redundant, specialized role in EVT specification that cannot be compensated by CREBBP. Alternatively, the apparent asymmetry may reflect differences in endogenous expression level and the combined functional contributions of these co-activators rather than true functional specialization. Distinguishing between these models will be critical for defining how KAT-mediated transcriptional regulation controls human trophoblast development.

In this study, we investigated the functional relationship between CREBBP and EP300 during human trophoblast stem cell differentiation, with a particular focus on EVT formation. By examining the effects of individual and combined perturbation of these co-activators, we sought to determine whether CREBBP and EP300 function redundantly, cooperatively, or through distinct mechanisms during trophoblast differentiation. Our findings provide new insight into the transcriptional regulation of human trophoblast specification and clarify the respective contributions of these closely related co-activators to placental development.

## 2. Results

### 2.1. EP300 Is Required for Differentiation

**Figure 1:**
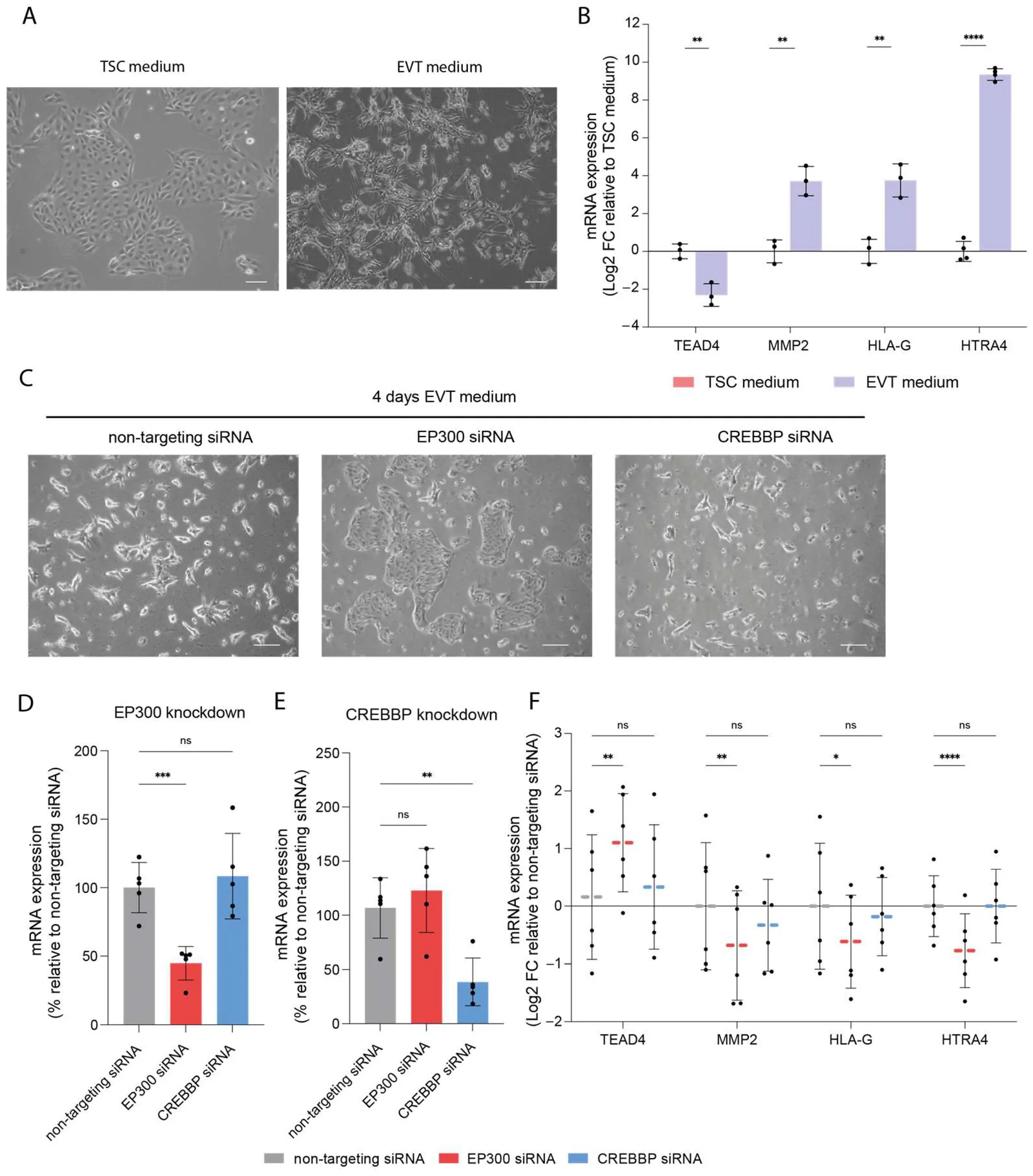
*EP300* knockdown inhibits EVT differentiation. (**A**) Phase-contrast microscopy images of TSCs cultured in TSC medium for 3 days or EVT medium for 5 days; (**B**) mRNA expression of the TSC marker *TEAD4*, EVT markers *MMP2*, *HLA-G* and *HTRA4* measured by qPCR. Cells in TSC medium were collected on day 3 and cells in EVT medium on day 5 after seeding. n = 3–4 independent experiments; (**C**) Phase-contrast microscopy images of TSCs transfected with non-targeting siRNA, *EP300* siRNA, or *CREBBP* siRNA and cultured in EVT medium for 4 days; (**D**) *EP300* and (**E**) *CREBBP* mRNA expression as a percentage relative to non-targeting siRNA control, measured by qPCR. n = 5 independent experiments; (**F**) mRNA expression of TSC marker *TEAD4*, EVT markers *MMP2*, *HLA-G*, and *HTRA4* in TSCs transfected with non-targeting siRNA, *EP300* siRNA, or *CREBBP* siRNA under EVT differentiation conditions. n = 6 independent experiments; Scale bars, 250 µm. Bars represent mean log2 fold-change ± SD. * *p* < 0.05, ** *p* < 0.01, *** *p* < 0.001, **** *p* < 0.0001; ns, not significant.

As previously described [17], we used TSCs from first-trimester placentas, and differentiation was induced by altering the composition of the culture medium. The TSCs displayed the characteristic cobblestone-like epithelial morphology, whereas EVTs exhibited a mesenchymal-like morphology (Figure 1A). Consistent with EVT differentiation, the expression of the trophoblast stem cell marker TEA domain transcription factor 4 (*TEAD4*) was decreased, while EVT-associated markers, including major histocompatibility complex class I, G (*HLA-G*), matrix metallopeptidase 2 (*MMP2*), HtrA serine peptidase 4 (*HTRA4*) [23,24], Transcription factor AP-2 gamma (*TFAP2C*) [25], Achaete-scute family bHLH transcription factor 2 (*ASCL2*) [26,27], Notum, palmitoleoyl-protein carboxylesterase (*NOTUM*) [28] and Integrin subunit alpha 1 (*ITGA1*) [25,29,30] were upregulated in EVTs (Figure 1B and Figure S1A).

Our previous work reported that knockdown of EP300 significantly impaired TSC differentiation into EVT, whereas knockdown of its closely related paralog CREBBP did not produce detectable defects in trophoblast differentiation [17]. We validated these findings in the present study (Figure 1C). qPCR confirmed that both *CREBBP* and *EP300* mRNA were reduced to approximately 40% of their endogenous levels following siRNA transfection (Figure 1D,E). This is in line with our previous study, in which the same knockdown strategy was validated at the protein level in TSCs. Consistent with the morphological observations, *EP300* knockdown resulted in a significant increase in the TSC marker *TEAD4*, indicating that stem cells retained their undifferentiated state. In contrast, *TEAD4* expression remained unchanged upon *CREBBP* knockdown (Figure 1F). Likewise, the EVT-associated markers *MMP2*, *HLA-G*, *HTRA4*, *TFAP2C*, *ASCL2*, *NOTUM* and *ITGA1* were markedly reduced in *EP300* knockdown cells but showed no significant difference in *CREBBP* knockdown cells (Figure 1F and Figure S1B).

### 2.2. Differential EP300 and CREBBP Expression Levels Suggest an Explanation for Their Asymmetric Effects on EVT Differentiation

**Figure 2:**
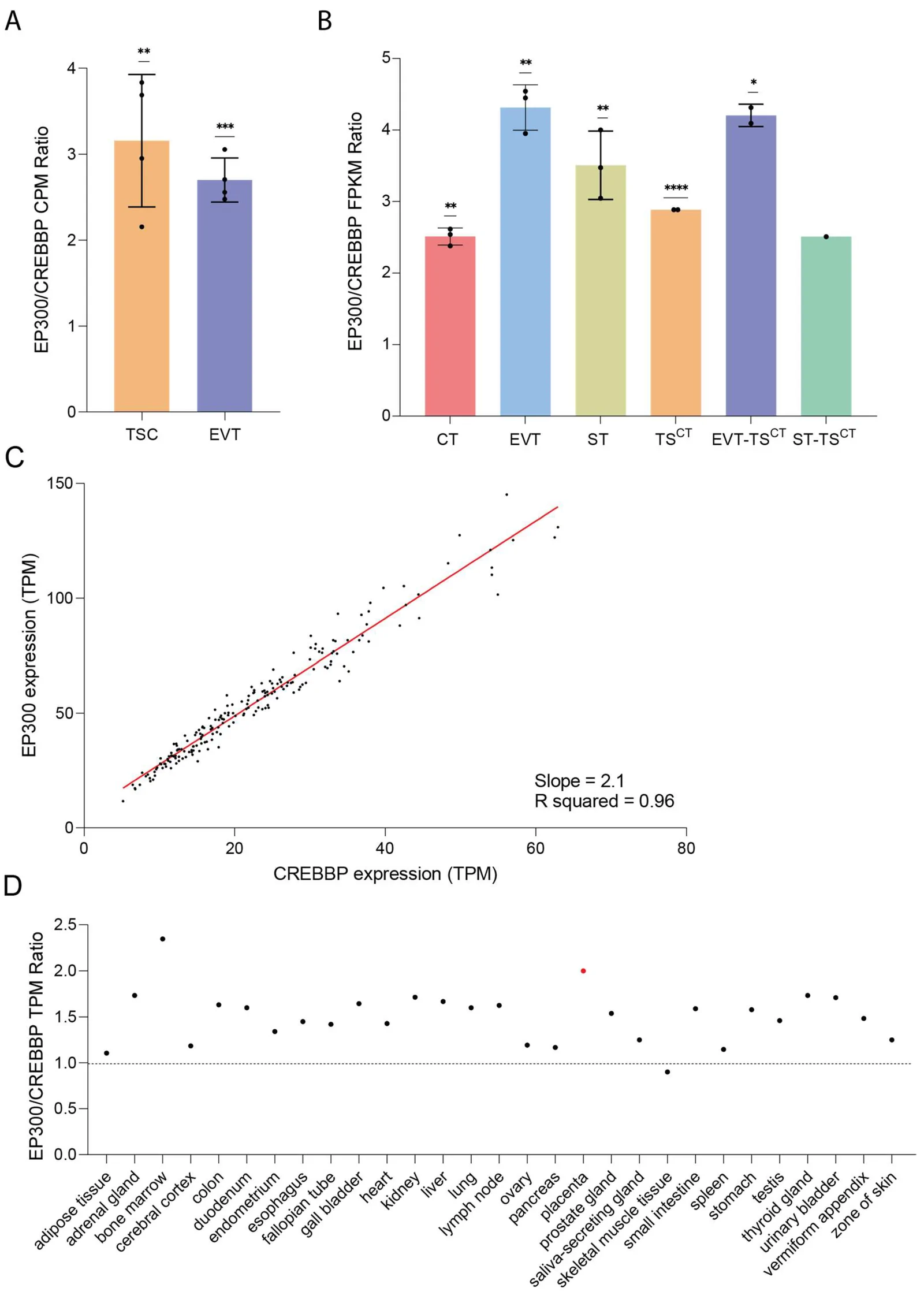
*EP300* mRNA is expressed at higher levels than *CREBBP* mRNA in placenta. (**A**) *EP300/CREBBP* ratio in TSC and EVT conditions at Day 4 of differentiation, calculated from batch-corrected counts per million (CPM) derived from RNA sequencing. n = 4 independent experiments; (**B**) *EP300/CREBBP* ratio in primary trophoblast samples and trophoblast stem cell derived cultures, calculated from fragments per kilobase of transcript per million mapped reads (FPKM) reported by Okae et al. [30]. CT, EVTs, and syncytiotrophoblasts (STs) represent primary trophoblast populations, whereas TS^CT^, EVT-TS^CT^, ST-TS^CT^ represent trophoblast stem cell-derived cultures as annotated in the original data set. Each dot represents one sample; (**C**) Linear regression analysis of *EP300* and *CREBBP* mRNA expression across 200 human term placenta samples based on analysis of a publicly available RNA-sequencing data set (NCBI BioProject PRJNA358255). Each dot represents an individual sample, with expression levels shown as transcripts per million (TPM). A strong positive correlation was observed (r^2^ = 0.96; slope = 2.1), with *EP300* consistently expressed at higher levels than *CREBBP*; (**D**) *EP300/CREBBP* expression ratios across human tissues based on RNA-seq data from EMBL-EBI Expression Atlas (E-MTAB-2836). Ratios were calculated from TPM values. Each dot represents a tissue type. The horizontal line indicates a ratio of 1, corresponding to equal expression of *EP300* and *CREBBP*. The placenta is highlighted in red; data are presented as mean ± SD. * *p* < 0.05, ** *p* < 0.01, *** *p* < 0.001, **** *p* < 0.0001.

Given the close similarity between CREBBP and EP300, the absence of a detectable differentiation phenotype following *CREBBP* knockdown was unexpected. To determine whether differences in endogenous expression could contribute to this observation, we analyzed RNA-sequencing data from TSCs and EVTs and found that *CREBBP* mRNA expression was approximately threefold lower than that of *EP300* in both conditions (Figure 2A). Expression values reported by Okae et al. revealed that *EP300* and *CREBBP* are expressed across all cytotrophoblasts (CTs), EVTs, and STBs [30]. *EP300* mRNA expression was consistently higher than that of *CREBBP* in these cells (Figure 2B). Furthermore, analysis of a large placenta RNA-sequencing dataset (NCBI BioProject PRJNA358255) revealed a strong linear relationship between *EP300* and *CREBBP* mRNA expression, with *EP300* expression consistently approximately 2-fold higher *CREBBP* expression across all samples (Figure 2C). To assess whether this expression imbalance extends beyond placenta, *EP300/CREBBP* expression ratios were calculated from the EMBL-EBI Expression Atlas for tissue expression data [31]. *EP300* expression exceeded *CREBBP* expression in the majority of human tissues, including placenta (Figure 2D). This expression imbalance was further supported at the protein level, as integrated placental protein abundance estimates from PaxDb indicated approximately twofold higher EP300 abundance than CREBBP (0.243 vs. 0.119 ppm; EP300/CREBBP ratio = 2.04) [32]. These findings suggest that the absence of an observable phenotype following *CREBBP* knockdown may reflect its substantially lower endogenous expression level relative to *EP300*. This raises the possibility that trophoblast differentiation efficiency depends on the combined levels of CREBBP and EP300, rather than those of EP300 alone. Based on these observations, we propose that CREBBP’s contribution to trophoblast differentiation becomes functionally apparent only under conditions of reduced EP300 activity or elevated CREBBP expression, consistent with its lower endogenous abundance.

### 2.3. Lower CREBBP Levels Increase TSC Sensitivity to A-485

**Figure 3:**
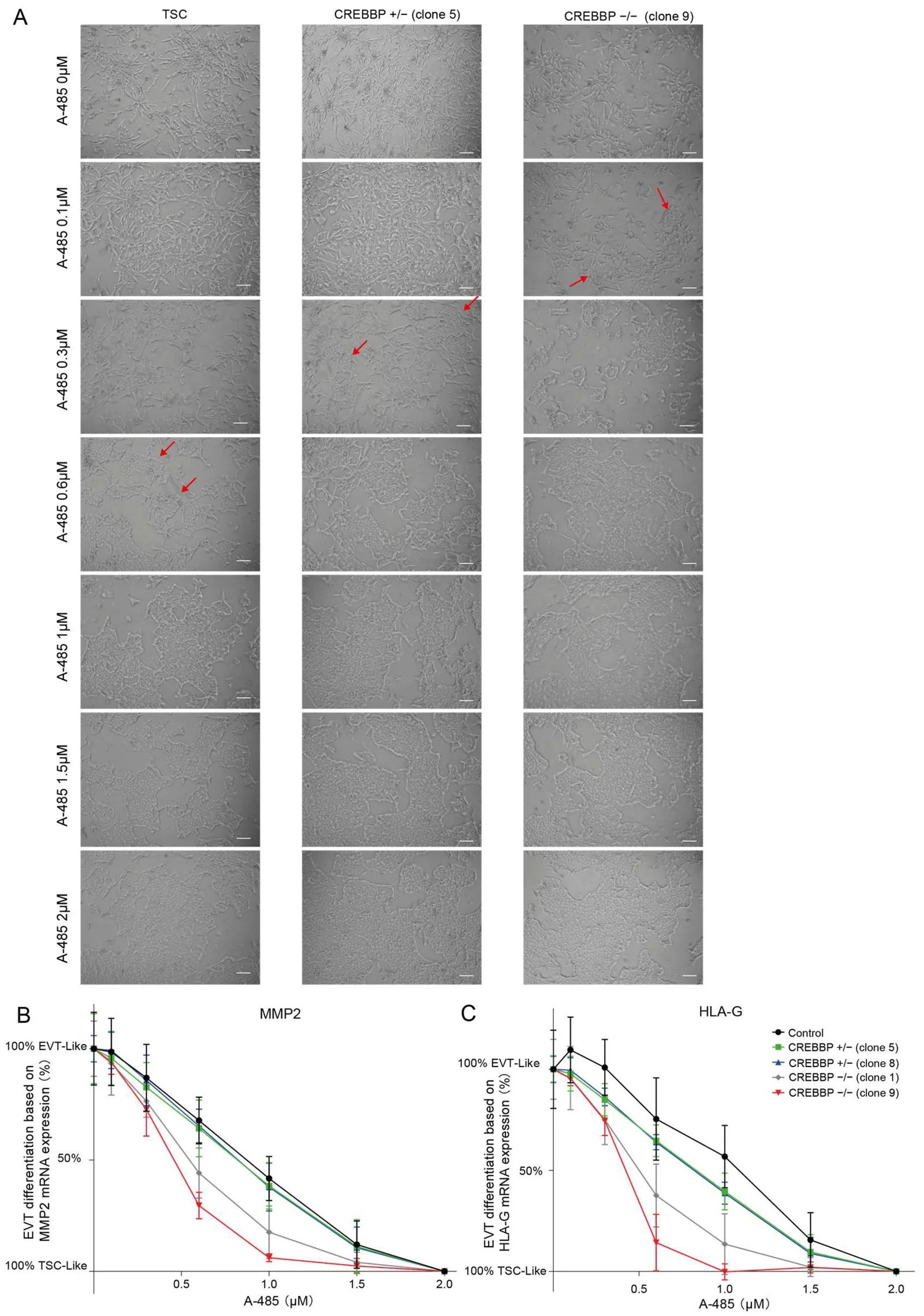
Reduced CREBBP levels increase TSC sensitivity to A-485-mediated inhibition of EVT differentiation. (**A**) Phase-contrast microscopy images of control TSCs, *CREBBP* heterozygous (clone 5), and *CREBBP* homozygous (clone 9) knockout cells cultured in EVT medium supplemented with increasing concentrations of the CREBBP/EP300 inhibitor A-485 for 6 days. Red arrows indicate patches with TSC-like morphology. Scale bars, 100 µm; Normalized (**B**) *MMP2* and (**C**) *HLA-G* mRNA expression in control TSCs, *CREBBP* +/−, and *CREBBP* −/− mutant cells treated with increasing concentrations of A-485 under EVT differentiation conditions. Expression is shown as a percentage relative to 100% maximal EVT-like and 100% TSC-like reference values. Data represent mean ± SD; n = 3 independent experiments. Statistical analysis was performed using two-way ANOVA with genotype and A-485 concentration as factors. For MMP2 (*p* < 0.01) and HLA-G (*p* < 0.0001), genotype was significantly different.

**Figure 4:**
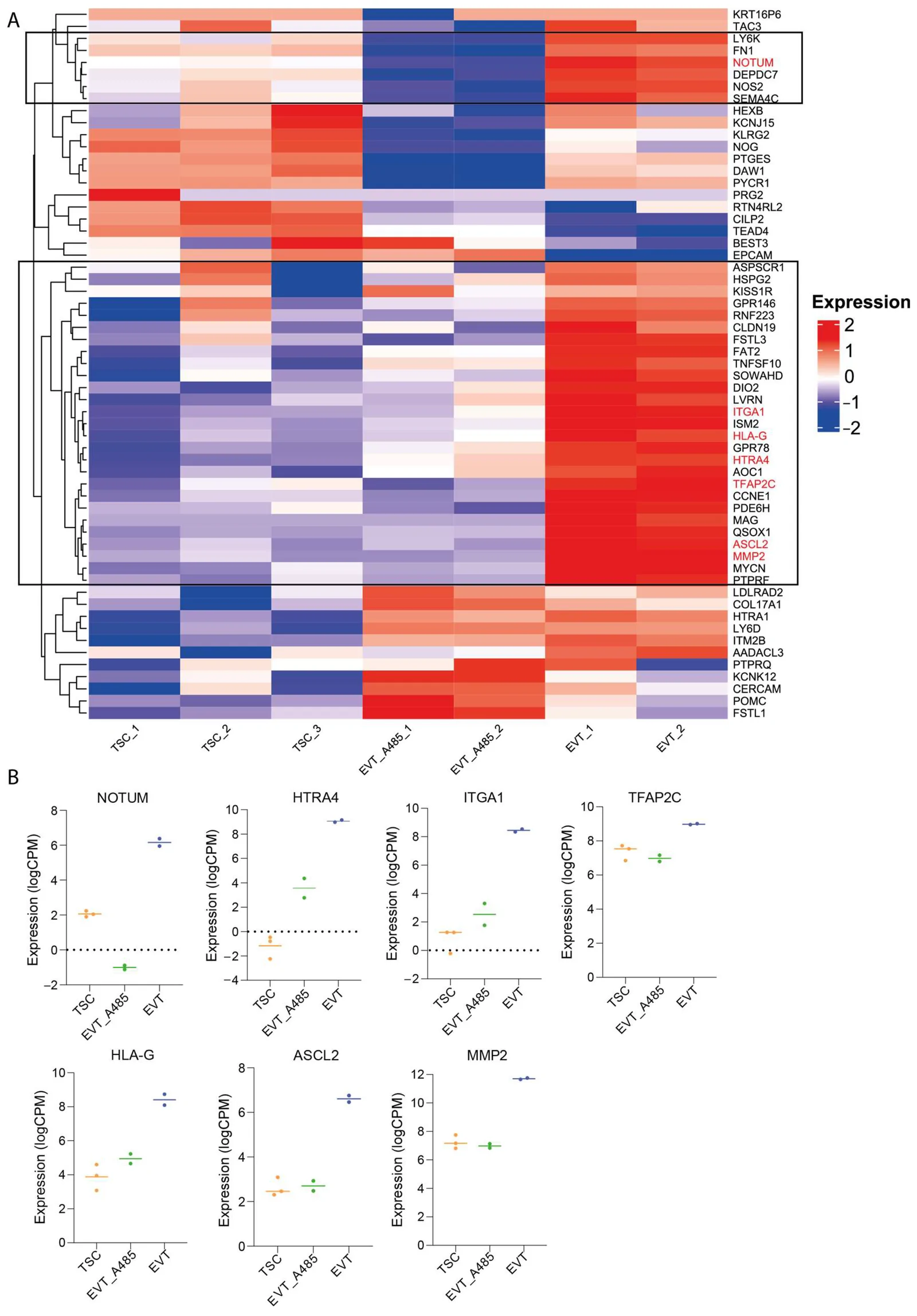
Heatmap of selected trophoblast-associated marker genes in TSCs, A-485-treated cells and EVT-differentiated cells. (**A**) Heatmap showing scaled expression of selected trophoblast-associated genes in TSCs, A-485-treated cells and EVT-differentiated cells. Columns represent individual samples: TSC_1–3, A485_1–2 and EVT_1–2. Genes selected for additional qPCR analysis are highlighted in red. The boxed region highlights a group of genes with high expression in EVT-differentiated samples and lower expression in TSC and A-485-treated samples. Expression values are scaled by gene and displayed from low expression in blue to high expression in red. (**B**) RNA-seq expression levels of selected genes used for additional qPCR analysis. Expression is shown as logCPM for TSC, A-485-treated and EVT-differentiated samples. Dots represent individual samples and horizontal lines indicate the mean.

The selective CREBBP/EP300 acetyltransferase inhibitor A-485 [33] has previously been shown to block EVT differentiation [17]. Consistent with this, A-485-treated cells showed impaired acquisition of the elongated, mesenchymal morphology characteristic of EVTs (Figure 3A). This effect was also reflected at the transcript level, as RNA-seq analysis showed reduced expression of many EVT-associated genes in A-485-treated samples compared with EVT-differentiated controls (Figure 4).

To test whether the contribution of CREBBP to trophoblast differentiation is masked by its lower endogenous expression, we generated heterozygous and homozygous *CREBBP* knockout TSCs by introducing premature stop codons through CRISPR/Cas9-mediated mutagenesis [17] (Figure S2B). These cells were then treated with A-485 under EVT-inducing conditions for 6 days.

Increasing concentrations of A-485 progressively impaired EVT differentiation, preventing cells from acquiring the elongated, mesenchymal morphology characteristic of EVTs and resulting in the retention of cobblestone-like TSC morphology and the formation of compact patches. This inhibitory effect shifted to lower A-485 concentrations as CREBBP levels decreased. Control TSCs began to exhibit patch-like morphology at 0.6 µM A-485, whereas heterozygous *CREBBP* knockout TSCs showed a similar response at 0.3 µM. Homozygous knockout TSCs displayed morphological inhibition at concentrations as low as 0.1 µM (Figure 3A and Figure S2A). Consistent with the morphological phenotype, qPCR analysis showed that expression of the EVT-associated markers *HLA-G*, *MMP2*, *ITGA1* and *NOTUM* progressively decreased with decreasing CREBBP gene copy number levels, while *HTRA4*, *ASCL2* and *TFAP2C* showed a similar trend (Figure 3B,C and Figure S3, Table S2).

These observations indicate that although CREBBP is expressed at lower levels than EP300, it contributes to EVT differentiation when EP300 activity is partially inhibited. The increased sensitivity of *CREBBP*-deficient TSCs to A-485 supports a functional contribution of CREBBP to trophoblast differentiation.

### 2.4. EVT Differentiation Is More Sensitive to A-485 upon EP300 Knockdown than CREBBP Knockdown

To directly compare the contributions of CREBBP and EP300 to EVT differentiation, we assessed the sensitivity of siRNA-mediated knockdown TSCs to A-485. Because siRNA provides only transient suppression, cells were collected on day 4 of EVT differentiation, when the first signs of mesenchymal morphology typically emerge. TSCs were transfected with *CREBBP* siRNA, *EP300* siRNA, or non-targeting siRNA as a control, and EVT differentiation was initiated the following day.

*EP300* knockdown TSCs exhibited the strongest inhibition of EVT differentiation in response to A-485, displaying impaired mesenchymal transition even in the absence of A-485. *CREBBP* knockdown TSCs showed an intermediate sensitivity, with patch-like inhibition appearing at approximately 0.3 µM A-485. In contrast, control TSCs began to exhibit comparable inhibition only at 0.6 µM (Figure S4). These results indicate that EP300 depletion increases sensitivity to A-485 more strongly than CREBBP depletion, while both perturbations enhance susceptibility relative to control cells.

Overall, reduced levels of either CREBBP or EP300 increased TSC susceptibility to A-485, supporting the notion that both co-activators contribute to EVT differentiation.

### 2.5. CREBBP Overexpression Rescues the Differentiation Defect Induced by EP300 Knockdown

**Figure 5:**
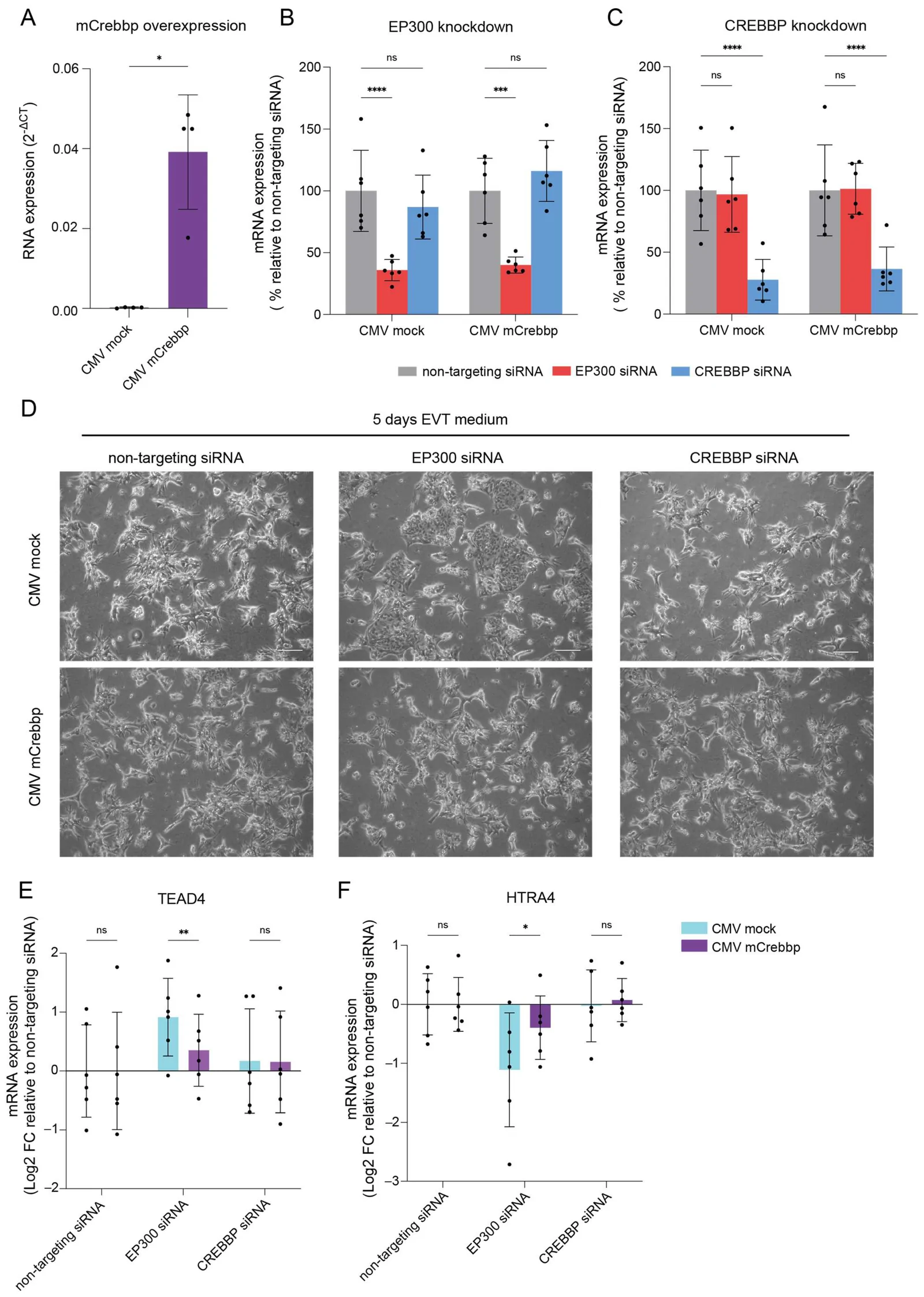
*mCrebbp* overexpression rescues the inhibitory effect of *EP300* knockdown on EVT differentiation. (**A**) *mCrebbp* mRNA expression in CMV mock-transduced and CMV *mCrebbp*-transduced cells measured by qPCR, confirming the transgene expression. Data represent mean ± SD; n = 4 independent experiments; (**B**) *EP300* and (**C**) *CREBBP* mRNA expression as a percentage relative to non-targeting siRNA control in CMV mock-transduced and CMV *mCrebbp*-transduced cells, measured by qPCR. Data represent mean ± SD; n = 6 independent experiments; (**D**) Phase-contrast microscopy images of TSCs transduced with CMV mock or a CMV promoter-driven *mCrebbp* expression (CMV *mCrebbp*) construct and subsequently transfected with non-targeting siRNA, *EP300* siRNA, or *CREBBP* siRNA, cultured in EVT medium for 5 days. Scale bars, 250 µm; (**E**) *TEAD4* and (**F**) *HTRA4* mRNA expression shown as Log2 FC relative to non-targeting siRNA control in CMV mock-transduced and CMV *mCrebbp*-transduced cells under EVT differentiation conditions for 5 days. Bars represent mean log2 fold-change ± SD. n = 6 independent experiments; * *p* < 0.05, ** *p* < 0.01, *** *p* < 0.001, **** *p* < 0.0001; ns, not significant.

To determine whether CREBBP can compensate for reduced EP300 activity, we performed rescue experiments in which *CREBBP* expression was upregulated in *EP300* knockdown TSCs. Mouse *Crebbp* (*mCrebbp*) was overexpressed using a CMV promoter-driven *mCrebbp* cDNA construct (CMV *mCrebbp*), with empty vector-transduced cells (CMV Mock) serving as controls. Following transduction, human *CREBBP* and *EP300* were knocked down using siRNA transfection. In *mCrebbp*-transduced cells (*mCrebbp* CMV), *mCrebbp* mRNA levels were markedly increased (Figure 5A), and efficient knockdown of *CREBBP* and *EP300* was confirmed (Figure 5B,C). As expected, *EP300* knockdown in CMV Mock cells led to characteristic patch-like morphology and impaired EVT differentiation. In contrast, *CREBBP* overexpression partially rescued this phenotype, with cells exhibiting some level of mesenchymal differentiation and almost no patch formation (Figure 5D and Figure S5), indicating that elevated CREBBP levels can restore EVT differentiation despite reduced EP300 expression. Moreover, *CREBBP* overexpression reduced *TEAD4* expression (Figure 5E) and increased *HTRA4*, *ITGA1* and *NOTUM* expression during EVT differentiation, while *ASCL2* and *TFAP2C* showed a non-significant upward trend (Figure 5F and Figure S6). Although the magnitude of these transcriptional changes was modest, they were consistent with the observed morphological rescue and support a partial restoration of EVT differentiation.

Together, these findings suggest that increased CREBBP can partially mitigate the differentiation defect caused by *EP300* knockdown, supporting a model in which CREBBP and EP300 jointly contribute to trophoblast differentiation.

## 3. Discussion

In this study, we show that CREBBP contributes to EVT differentiation despite its lower endogenous expression, whereas EP300 has a predominant role under physiological conditions. Reduced CREBBP levels increased cellular sensitivity to pharmacological inhibition, and elevated CREBBP expression partially rescued differentiation defects caused by EP300 depletion. These findings indicate that the apparent asymmetry between CREBBP and EP300 can be explained, at least in part, by differences in their endogenous expression levels, and support a model in which CREBBP and EP300 jointly contribute to EVT differentiation.

**Figure 6:**
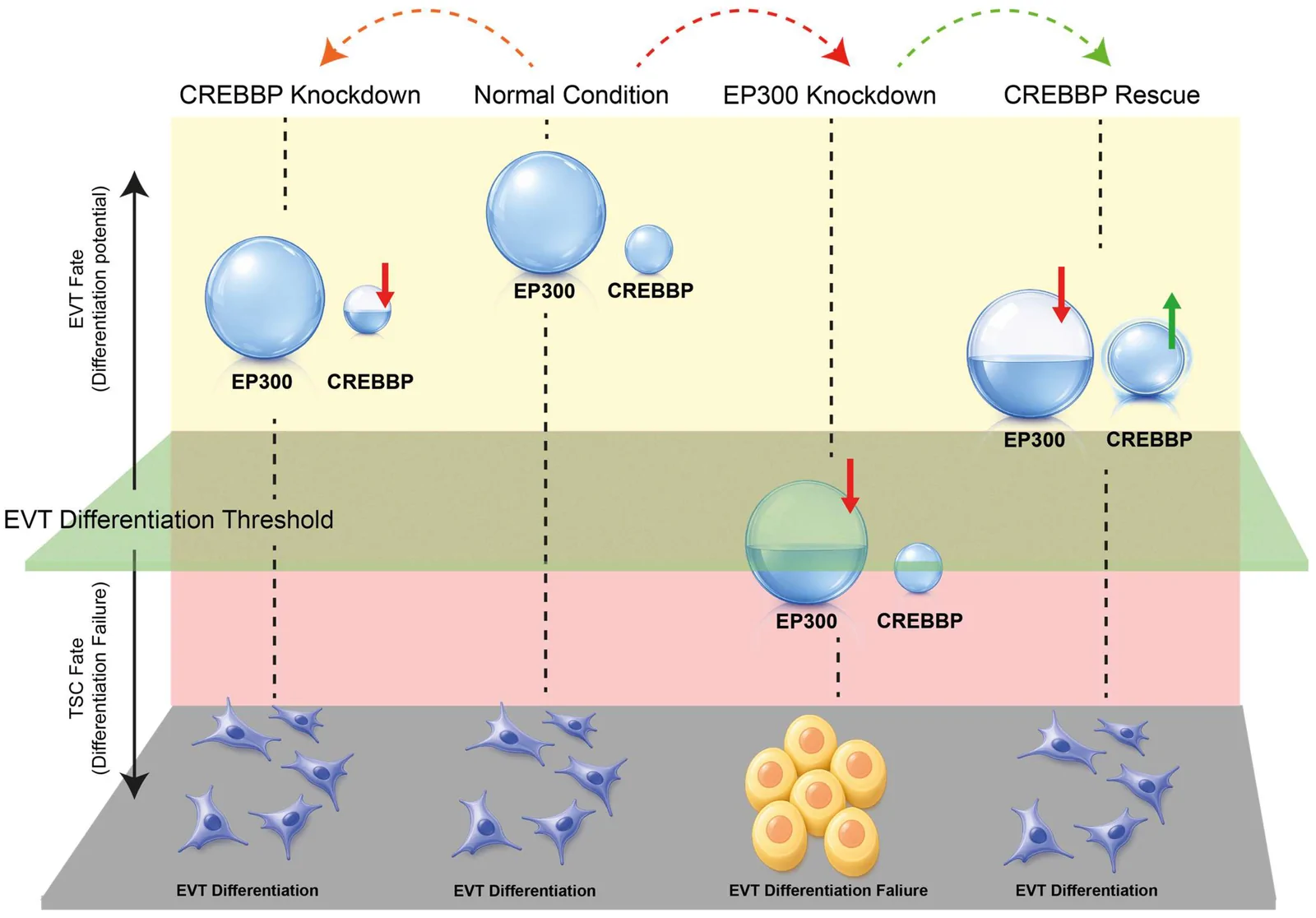
Proposed model of combined CREBBP and EP300 levels during EVT differentiation. Under normal conditions, EP300 provides the major contribution to the total functional activity of EP300 and CREBBP, whereas CREBBP contributes to a lesser extent, resulting in activity levels above the threshold required for EVT differentiation. Depletion of EP300 reduces total activity below this threshold, leading to differentiation failure. In contrast, CREBBP depletion has only a limited effect because EP300 maintains sufficient activity to support differentiation. Increasing CREBBP expression partially restores total activity in EP300-deficient cells, enabling recovery of EVT differentiation. The green shaded threshold region indicates the minimum activity level required for EVT differentiation.

*EP300* knockdown severely impaired EVT differentiation, whereas *CREBBP* depletion alone had minimal effect, consistent with previous studies [17]. We found that *CREBBP* is expressed at approximately one-third of the level of *EP300* in both TSCs and EVTs, providing a possible explanation for this asymmetry. Under baseline conditions, the lower endogenous expression of CREBBP relative to EP300 suggests that it contributes less than EP300 to CREBBP/EP300-dependent regulation for EVT differentiation. Therefore, partial depletion of CREBBP may have a limited effect because EP300 remains sufficient to support EVT differentiation (Figure 6). In contrast, EP300 depletion markedly reduces CREBBP/EP300-dependent function and leads to a clear differentiation defect. Consistent with this model, EP300 and CREBBP have been reported to share many genomic binding sites and transcriptional targets in other cellular contexts [34], suggesting that differentiation competence may depend primarily on total CREBBP/EP300 levels rather than paralog-specific functions [22].

Pharmacological inhibition further supports a threshold-like requirement for CREBBP/EP300 activity. Reducing CREBBP levels sensitized cells to A-485 in a stepwise manner, and EP300 depletion further enhanced this effect, indicating CREBBP and EP300 jointly contribute to EVT differentiation. These findings suggest that EVT differentiation depends on maintaining a minimum level of CREBBP/EP300 activity, below which differentiation efficiency is compromised. Similar functional cooperation between paralogs chromatin regulators has been described in other biological systems, where combined perturbation produces stronger phenotypes than single-gene disruption [35,36]. For example, in MLL-rearranged acute myeloid leukemia models, deletion of either CREBBP or EP300 suppressed proliferation and colony formation, and pharmacological inhibition further highlighted the importance of total co-activator function [35].

Notably, *Crebbp* overexpression partially restored EVT differentiation in *EP300*-depleted cells, indicating that it is intrinsically capable of supporting EVT differentiation when expressed at sufficient levels. This argues against strict paralog-specific specialization and instead suggests that EP300’s apparent dominance primarily reflects its higher endogenous expression rather than complete functional specialization. Given their conserved histone acetyltransferase (HAT) domains and largely overlapping substrate specificity [13], both proteins likely possess similar catalytic potential. The greater contribution of EP300 to EVT differentiation may therefore primarily reflect its higher endogenous abundance rather than fundamental functional differences between the two paralogs.

The downstream mechanisms through which CREBBP and EP300 regulate EVT differentiation remain incompletely understood. One possible explanation is their acetyltransferase activity, as CREBBP and EP300 are major lysine acetyltransferases that catalyze histone H3 lysine 27 acetylation (H3K27ac) at active regulatory elements [15]. H3K27ac is a canonical mark of active enhancers that defines transcriptionally competent regulatory regions [37,38]. During trophoblast differentiation, the enhancer landscape undergoes dynamic remodeling accompanied by changes in H3K27ac occupancy [18,25,39]. Consistent with this, EP300 binding has been reported to co-localize with trophoblast-specific enhancers, suggesting a potential role in activating EVT lineage-specific transcriptional programs [40]. Together, these observations suggest that CREBBP/EP300 may influence EVT differentiation through effects on enhancer activity and downstream transcriptional programs. Although our findings identify overall CREBBP/EP300 activity as a major determinant of EVT differentiation, the signaling pathways acting downstream remain to be defined. Previous studies indicate that EP300 influences several signaling pathways relevant to trophoblast biology, likely through both chromatin-level and non-histone acetylation mechanisms. For instance, EP300 depletion upregulates epidermal growth factor receptor (EGFR) ligand expression [17], consistent with a role in restraining EGF-driven cell proliferation during differentiation. Given the importance of Hippo [41,42], transforming growth factor-β (TGF-β) [43], Wnt signaling, and epithelial-to-mesenchymal transition (EMT)-related regulators [44] in trophoblast lineage specification, future studies should clarify how CREBBP/EP300 levels and total acetyltransferase activity affect enhancer activity, transcription factor networks and signaling cascades during EVT differentiation.

Although overexpression of *mCrebbp* resulted in a clear increase in mRNA levels, no obvious corresponding increase in CREBBP levels was detected by immunoblotting. Despite this, Crebbp overexpression partially rescued the differentiation defects caused by *EP300* knockdown, as evidenced by morphological recovery and restoration of EVT marker expression. Discrepancies between mRNA abundance and protein levels are common due to post-transcriptional regulation and protein turnover [45,46]. Moreover, the rescue phenotype suggests that biologically relevant CREBBP activity was increased despite the absence of a detectable change in total protein abundance. Given the high structural similarity and partially redundant co-activator functions of CREBBP and EP300 [14], even a relatively small increase in CREBBP activity may be sufficient to partially restore total CREBBP/EP300 function and thereby support EVT differentiation.

The possible broader developmental relevance of CREBBP/EP300 function should be interpreted cautiously. Pathogenic variants in both *CREBBP* and *EP300* cause Rubinstein-Taybi syndrome (RSTS), a rare congenital disorder characterized by intellectual disability and distinctive physical features [47]. RSTS is genetically heterogeneous, with most cases caused by mutations in *CREBBP* (KAT3A; RSTS type 1), whereas mutations in *EP300* (KAT3B; RSTS type 2) are less common [48,49,50]. Specifically, *EP300*-associated RSTS has been reported to display a very high incidence of pregnancy-related complications, including fetal growth restriction and pre-eclampsia [51]. Together with our current findings we speculate that the RSTS type 2 specific pregnancy complications may be the result of the dominant role of EP300 levels in trophoblast differentiation, while the impact of a reduced CREBBP expression in RSTS type 2 may not have such an impact on the placenta. However, additional studies are required to further support or discard this hypothesis.

The current study has certainly limitations, we have used a single TSC line, although we have shown previously that the effects of CREBBP and EP300 is shared by additional independently derived TSC lines [17]. In addition, CREBBP and EP300 were mainly assessed at the transcript level, while the final function of CREBBP and EP300 is at a complex interplay of protein acetyltransferase activity, protein-protein interactions and subcellular location. The direct genomic targets of CREBBP and EP300 during EVT differentiation remain unclear. Although our findings support a model of CREBBP/EP300 function, the underlying chromatin and transcriptional mechanisms were not directly examined. Future chromatin profiling studies will be required to identify the regulatory elements and target genes through which CREBBP/EP300-dependent function regulates EVT differentiation. In addition, the use of mouse Crebbp may have limited the rescue ability. Although the mCrebbp protein is 96% identical at the amino acid level to the human CREBBP, there still may be difference in function that would make mCrebbp not fully capable of replacing human CREBBP.

In summary, our findings demonstrate that EVT differentiation is highly sensitive to combined levels of CREBBP/EP300. EP300 serves as the dominant contributor under physiological conditions because of its higher expression levels, whereas CREBBP is capable of compensating when sufficiently abundant. These findings underscore the importance of quantitative regulation of epigenetic co-activators in trophoblast lineage progression and support a model in which both KAT3 co-activators jointly regulate human placental development.

## 4. Materials and Methods

### 4.1. Cell Culture and Maintenance

Human TSCs used in this study were derived from the first-trimester placenta-derived TSC_1 line previously established and characterized [17]. This line was generated from first-trimester placental tissue and has been shown to maintain trophoblast stem cell morphology, marker expression and the capacity to differentiate into EVT, STB and trophoblast organoid models. All experiments in the present study were performed using TSC_1 or genetically modified derivatives of this line. Although the cell source was the same as in the previous study, cell expansion and differentiation for the present experiments were performed under the culture conditions described below.

To propagate TSCs, these cells were seeded on culture plates coated with 5 μg/mL mouse collagen IV in TSC medium composed of DMEM/F-12 with GlutaMAX (Gibco, Waltham, MA, USA, 31331), supplemented with 0.8 mM Valproic Acid, 1% ITS-X Supplement, 1% Knockout Serum Replacement, 0.15% Bovine serum albumin solution, 0.5% Penicillin-Streptomycin (Gibco, 15140122), 57 μg/mL L-ascorbic acid, 5 μM A83-01, 2.5 μM Y-27632, 50 ng/mL Human Recombinant EGF, and 2 μM CHIR99021 (see Table S3 for details of chemicals used in cell culture). Cells were maintained at 37 °C in a humidified incubator with 5% CO_2_. When cultures reached 70–80% confluence, cells were dissociated with TrypLE for 15 min at 37 °C and passaged. TSCs between passages 10 and 30 were used for all experiments.

HEK293T cells were maintained in high-glucose DMEM supplemented with 10% Fetal Bovine Serum, 6 mM L-Glutamine, 0.1 mM MEM non-essential amino acids, 1% Penicillin-Streptomycin (see Table S3 for details of chemicals used in cell culture). Cells were cultured at 37 °C in 5% CO_2_ and passaged using 0.25% Trypsin-EDTA when they reached full confluence, typically every 3–4 days.

### 4.2. EVT Differentiation

To differentiate TSCs to EVTs, TSCs were seeded on culture plates coated with 1 µg/mL mouse collagen IV in EVT medium A, which consisted of DMEM/F-12 with GlutaMAX supplemented with 0.1 mM 2-Mercaptoethanol, 0.3% Bovine serum albumin solution, 1% ITS-X Supplement, 0.5% Penicillin-Streptomycin, 7.5 µM A83-01, 2.5 µM Y-27632, 100 ng/mL Human Recombinant Heregulin-beta 1, 4% Knockout Serum Replacement, and 2% growth factor-reduced (GFR) Matrigel (see Table S3 for details of chemicals used in cell culture) on day 1. At differentiation day 3, medium was replaced with EVT medium B (EVT medium A supplemented with 0.5% GFR Matrigel and without Human Recombinant Heregulin-beta 1). Unless otherwise indicated, EVTs were harvested on day 6 of differentiation for subsequent analyses. Morphological changes and marker expression were monitored at defined time points throughout the differentiation process.

### 4.3. RNA Isolation and Quantitative Real-Time PCR (RT-qPCR)

Monolayer TSCs were washed with PBS and lysed directly in the culture plate using RLT buffer (RNeasy Plus Mini Kit, QIAGEN, 74136, Hilden, Germany) containing 1% 2-mercaptoethanol (Sigma-Aldrich, 63689, St. Louis, MO, USA). Total RNA was extracted following the manufacturer’s protocol (RNeasy Mini Kit, QIAGEN, 74136). First-strand cDNA was synthesized from 1 µg of total RNA using 500 ng random primers (Promega, C1181, Madison, WI, USA), 1× M-MLV Reverse Transcriptase Reaction Buffer (Promega, M531A), 200 U M-MLV Reverse Transcriptase (Promega, M170B), 10 mM dNTP mix (Thermo Scientific, R0192, Waltham, MA, USA), and 25 U RNasin Ribonuclease Inhibitor (Promega, N251B) in a 25 µL reaction.

qPCR was performed using 2.5 µL of 3× diluted cDNA, 0.5 µM primers (Table S5 for details), and 1× LightCycler 480 SYBR Green I Master (Roche Diagnostics, 04887352001, Rotkreuz, Switzerland) in a total volume of 10 µL on a LightCycler 480 system (Roche Diagnostics). Relative gene expression was calculated using the comparative cycle threshold (ΔΔCt) method [52], normalized to the reference genes *GUSB*, *EEF2* and *PSMD4*, and expressed relative to the geometric mean of control samples.

### 4.4. Small Interfering RNA (siRNA) Transfection

TSCs were seeded at 1 × 10^5^ cells per well in a 6-well plate in 2.5 mL of TSC medium without Penicillin-Streptomycin and incubated overnight. Cells were transfected the following day with 10 nM siRNA targeting *CREBBP*, *EP300*, or a non-targeting control (see Table S4 for product details) using Lipofectamine RNAiMAX Transfection Reagent (Thermo Fisher Scientific, 13778150, Waltham, MA, USA) according to the manufacturer’s instructions. Briefly, siRNA and Lipofectamine RNAiMAX were each diluted in Opti-MEM I Reduced Serum Medium (Gibco, 31985062, Waltham, MA, USA), combined to form complexes, incubated for 5 min at room temperature, and added to cells.

Transfected cells were incubated at 37 °C with 5% CO_2_ for 24 h. Cells were then harvested for RNA isolation and qPCR analysis, or dissociated for initiation of EVT differentiation.

### 4.5. Transcriptome Sequencing

For the RNA-seq analysis shown in Figure 2A, total RNA was extracted and sequencing libraries were constructed using the KAPA RNA HyperPrep Kit with RiboErase (Roche, 8098140702). Paired-end sequencing (150 bp) was performed on an Illumina NovaSeq 6000 platform. Raw read quality was assessed with FastQC (v0.11.8) and FastQ Screen (v0.14.1), and adapter trimming was carried out using Trim Galore (v0.6.6). Trimmed reads were mapped to the human reference genome (GRCh38, Ensembl v107) with STAR (v2.7.1a), and output BAM files were indexed using Samtools (v1.11). Post-alignment quality metrics were evaluated using RSeQC (v5.0.1) and Qualimap (v2.3), and summarized with MultiQC (v1.13). Read counts at the gene level were quantified using featureCounts (v2.0.0), with multi-mapped reads excluded. All downstream analyses were performed in R (v4.5.1). Read counts were corrected for batch effects using ComBat-seq (sva v3.42.0) and normalized using edgeR (v4.6.3). *CREBBP* and *EP300* expression levels were extracted as CPM values.

For Figure 4, a subset of bulk RNA-seq samples from our previous study (GEO accession GSE282356) was reused in the present analysis, together with four additional samples from the same sequencing run that were not part of the original dataset (GEO accession GSE343715). For this analysis, Salmon (v1.9.0) was used to quantify transcript abundance against the GRCh38 transcriptome (Ensembl release 107), and the resulting files were imported into R (v4.6.0) using tximport (v1.32.0). Gene-level counts were normalized with edgeR (v4.10.0) and converted to log2 counts per million (logCPM).

For heatmap generation, genes from the DESCARTES fetal placenta EVT set (DESCARTES_FETAL_PLACENTA_EXTRAVILLOUS_TROPHOBLASTS) [53] were selected based on gene symbols and combined with *MMP2*, *ITGA1*, *TFAP2C*, *TEAD4*, and *EPCAM*. Hierarchical clustering and heatmap visualization were performed using ComplexHeatmap (v2.28.0).

### 4.6. A-485 Treatment

A-485 (MedChemExpress, HY-107455, Monmouth Junction, NJ, USA) was added to EVT differentiation medium on the first day at concentrations of 0.1, 0.3, 0.6, 1.0, 1.5, and 2 µM and added again whenever the medium was changed. Morphological changes were subsequently monitored.

### 4.7. CRISPR/Cas9-Mediated Mutagenesis

CRISPR/Cas9-mediated mutagenesis of *CREBBP* in TSCs was performed as described previously [17]. In brief, guide RNAs were cloned into the lentiCRISPR v2 vector (Addgene 52961, Watertown, MA, USA) and transfected into TSCs using X-tremeGENE HP DNA Transfection Reagent (Roche, 6366236001) at a 1:2 ratio. Transfected cells were selected with 2.5 µg/mL Puromycin (Merck, P8833, Darmstadt, Germany) for 2 days and then seeded at low density for clonal expansion. Successful mutant clones were validated by genomic sequencing and Western blotting [17] (Table S1 and Figure S2B).

### 4.8. Nuclear Protein Extraction and Western Blotting

Nuclear protein extraction and Western blotting were performed as described in our previous work [17]. In short, pelleted cells were incubated in hypotonic buffer on ice, followed by addition of NP-40 and centrifugation to collect nuclear fractions. Nuclear proteins were extracted using cell lysis buffer (Invitrogen, FNN0011, Waltham, MA, USA) supplemented with protease inhibitors (1 mM PMSF and 1× Protease Inhibitor Cocktail (Roche, 11836170001)), and protein concentrations were measured using a Pierce BCA Protein Assay Kit (Thermo Scientific, 23227). Equal amounts of nuclear protein (10 μg) were separated on NuPAGE 4–12% Bis-Tris gels (Invitrogen, NP0321BOX) and transferred to PVDF membranes (Sigma-Aldrich, IPFL00010). Membranes were blocked, incubated overnight with primary antibodies (see Table S6 for product details and dilutions), followed by IRDye 800CW-conjugated secondary antibody (LI-COR, 925-32211, Lincoln, NE, USA), and imaged using a LI-COR Odyssey system. Band intensities were quantified using Image Studio (v6.0) and normalized to Lamin B1.

### 4.9. Lentiviral Production

Lentiviral particles were generated in HEK293T cells using a standard three-plasmid packaging system. One day before transfection, HEK293T cells were seeded on Poly-L-lysine hydrobromide (Merck, P1274) coated plates and cultured to 85–95% confluency. For each well, 2.5 µg of plasmid containing the vector of interest, 364.7 ng pMD2.G (Addgene, 12259), 607.8 ng pMDLg/pRRE (Addgene, 12251) and 303.9 ng pRSV-Rev (Addgene, 12253) were mixed with 6 µL X-tremeGENE HP (Roche, 6366236001) in Opti-MEM I Reduced Serum Medium (Gibco, 31985062), adjusted to a final Opti-MEM concentration of 0.01 ng/µL. The transfection mixture was added directly to 2 mL of HEK293T culture medium.

Cells were incubated for 8 h to overnight at 37 °C before the medium was replaced with fresh HEK293T medium supplemented with 20 mM HEPES Buffer Solution (Gibco, 15630-056) and 4 mM Caffeine (SIGMA, C0750). Virus-containing supernatants were collected at 48 h and 96 h post-transfection, clarified, and mixed with Lenti-X Concentrator (Takara Bio, 631231, San Jose, CA, USA) at a 1:3 ratio (concentrator:supernatant). After incubation at 4 °C overnight, samples were centrifuged at 1500× *g* for 45 min at 4 °C.

The viral pellet was gently resuspended, aliquoted, and stored at −80 °C. Viral titers were determined prior to use.

### 4.10. mCREBBP cDNA Overexpression

The *mCrebbp* cDNA was first excised from pRc/RSV-mCBP-HA (Addgene, 16701) and cloned into an entry vector (Addgene, 17461). Using Gateway cloning, the *mCrebbp*-containing entry vector was subsequently recombined into the pLenti CMV Blast DEST lentiviral expression vector (Addgene, 17451). High-titer lentiviral particles carrying either the *mCrebbp* vector or the empty vector control were produced in HEK293T cells as described above and used to transduce TSCs at a multiplicity of infection (MOI) of 0.3. Following transduction, cells were selected with 2 µg/mL Blasticidin (Merck, 10205) for 4–6 days, with passaging when cultures reached ~80% confluence. Stable overexpression populations were expanded and validated by qPCR prior to use in downstream transfection and EVT differentiation experiments.

### 4.11. Microscopy

Cell morphology during EVT differentiation was monitored by phase-contrast microscopy using a Leica DMi8 (Leica Microsystems, Wetzlar, Germany). Images were acquired at defined time points under identical illumination and exposure settings for each experiment. Grayscale levels were uniformly adjusted in Leica Application Suite software (v3.4.2.18368) to improve contrast, and the same acquisition and post-processing parameters were applied to all samples.

### 4.12. Statistics

Data are presented as mean ± standard deviation (SD). Statistical analyses were performed using GraphPad Prism (v10.6.0). For comparisons between two matched groups, a paired two-tailed *t*-test was used. For comparisons of multiple groups against a single control, one-way repeated-measures ANOVA followed by Dunnett’s multiple comparisons test was performed. For ratio analyses, *EP300/CREBBP* expression ratios were calculated for each sample. Ratios were log2-transformed for statistical analysis and tested against a theoretical log2 ratio of 0 using one-sample *t*-tests, corresponding to equal expression of EP300 and CREBBP. Untransformed ratios are shown for visualization. For dose-response curve analysis, statistical significance was assessed using two-way ANOVA, with A-485 concentration and genotype as the two factors, followed by Tukey’s test for multiple comparisons. For all other comparisons, the Bonferroni correction was applied where appropriate. RT-qPCR data were analyzed and presented on a log2 scale. *p* values < 0.05 were considered statistically significant.

### 4.13. Generative AI

Generative AI tools were used to assist with minor language editing and rephrasing pieces of text to improve readability, and minor graphical components for the schematic illustration shown in Figure 6. The final text and schematic illustration were reviewed, edited, and approved by the authors. The schematic illustration was designed and assembled by the authors using Adobe Illustrator (v29.8.2).

## Data Availability

The original transcriptome sequencing data presented in the study are openly available in the Gene Expression Omnibus at GSE336656 and GSE343715.

## Supplementary Materials

The following supporting information can be downloaded at: https://www.mdpi.com/article/10.3390/ijms27177623/s1.

## Abbreviations

The following abbreviations are used in this manuscript:

EVTs	Extravillous trophoblast cells
TSCs	Trophoblast stem cells
CTB	Cytotrophoblast
STBs	Syncytiotrophoblasts
CREBBP	CREB-binding protein
EP300	E1A-binding protein p300
KATs	lysine acetyltransferases
TEAD4	TEA domain transcription factor 4
HLA-G	histocompatibility complex class I, G
MMP2	matrix metallopeptidase 2
HTRA4	HtrA serine peptidase 4
TFAP2C	Transcription factor AP-2 gamma
ASCL2	Achaete-scute family bHLH transcription factor 2
NOTUM	Notum, palmitoleoyl-protein carboxylesterase
ITGA1	Integrin subunit alpha 1
CPM	counts per million
FPKM	fragments per kilobase of transcript per million mapped reads
TPM	transcripts per millio
HAT	histone acetyltransferase
H3K27ac	histone H3 lysine 27 acetylation
EGFR	epidermal growth factor receptor
TGF-β	transforming growth factor-β
EMT	epithelial-to-mesenchymal transition
RSTS	Rubinstein-Taybi syndrome
