# Supplementary material for: Combined EP300 and CREBBP Expression Levels Regulate Human Trophoblast Differentiation
Source: Int J Mol Sci. 2026 Aug 25;27(17):7623. doi: 10.3390/ijms27177623 (PMC13565857; doi:10.3390/ijms27177623)
Supplement: Supplementary file 1 [file ijms-27-07623-s001.zip › ijms-4437066-supplementary.pdf]

Supplementary Information for

Combined EP300 and CREBBP expression levels regulate human trophoblast differentiation

Fangxu Lin [1], Remco Keijser [1] and Gijs Afink [1,*]

Reproductive Biology Laboratory, Amsterdam Reproduction and Development, Amsterdam UMC, University of Amsterdam, 1105 AZ Amsterdam, The Netherlands; l.lin@amsterdamumc.nl (F.L.); r.keijser@amsterdamumc.nl (R.K.)

* Correspondence: g.b.afink@amsterdamumc.nl

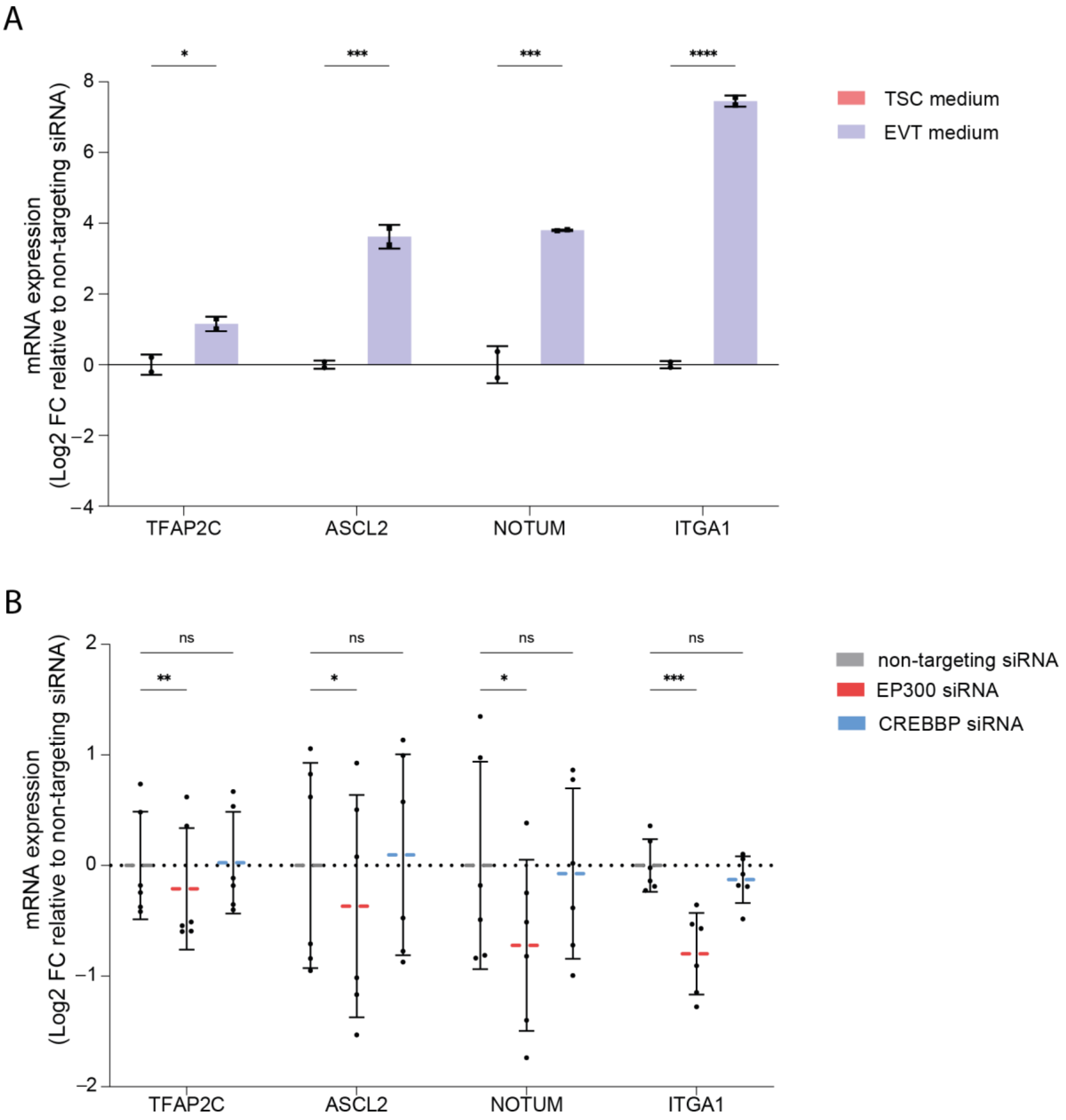

Figure S1. (related to Figure 1). Additional qPCR analysis of trophoblast marker expression during EVT differentiation and CREBBP/EP300 knockdown. (A) mRNA expression of EVT-associated markers *TFAP2C, ASCL2, NOTUM* and *ITGA1*, measured by qPCR. Cells in TSC medium were collected on day 3 and cells in EVT medium on day 6 after seeding. n = 2 independent experiments; (B) mRNA expression of *TFAP2C, ASCL2, NOTUM* and *ITGA1* in TSCs transfected with non-targeting siRNA, *EP300* siRNA, or *CREBBP* siRNA under EVT differentiation conditions. n = 6 independent experiments. Bars represent mean log2 fold-change ± SD . *$p < 0.05$, **$p < 0.01$, ***$p < 0.001$, ****$p < 0.0001$; ns, not significant.

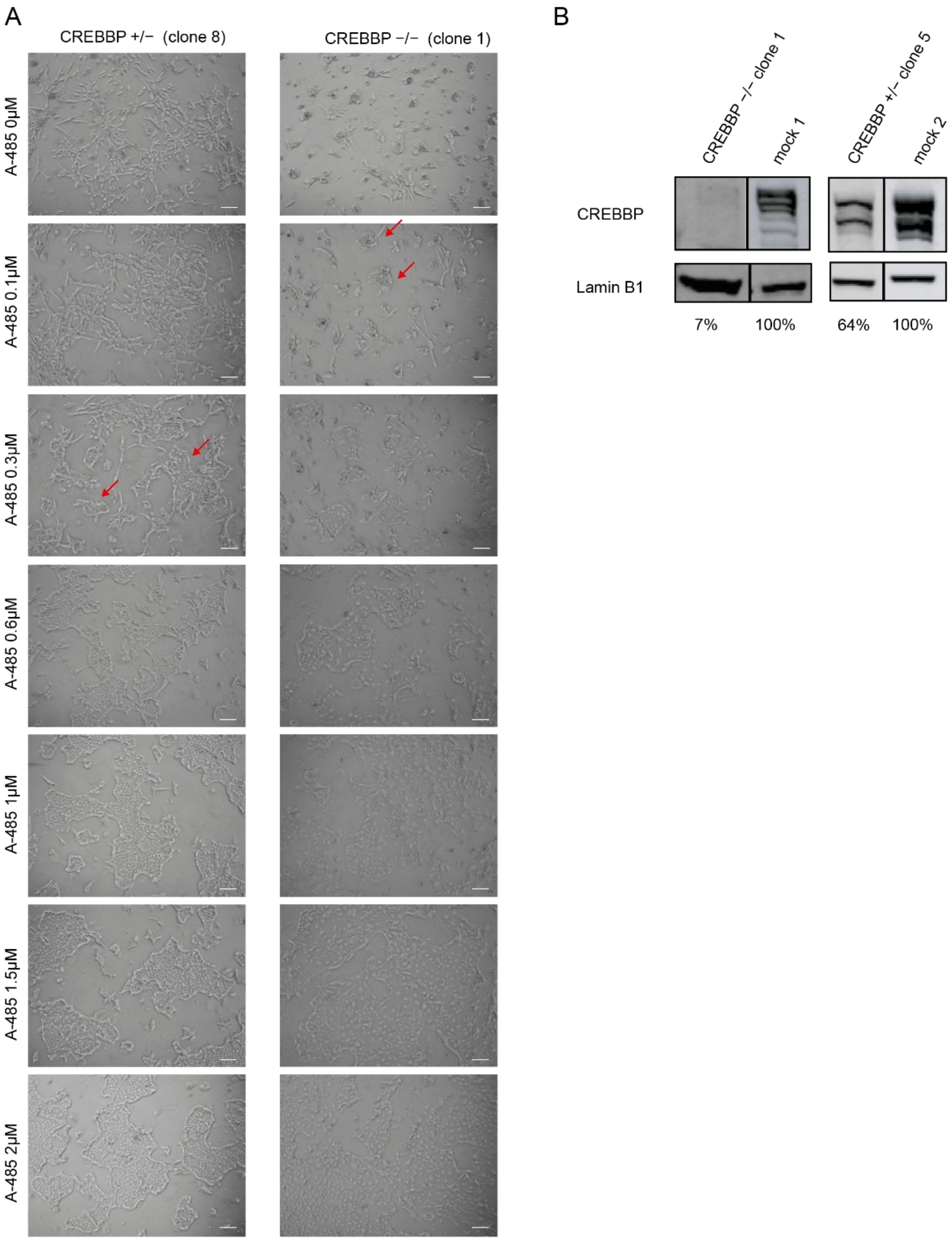

Figure S2. (related to Figure 3). *CREBBP*-deficient TSCs show increases sensitivity to A-485-mediated inhibition of EVT differentiation. (A) Phase-contrast microscopy images of control TSCs, *CREBBP* heterozygous (clone 8) and *CREBBP* homozygous (clone 1) knockout cells cultured in EVT medium supplemented with increasing concentrations of the CREBBP/EP300 inhibitor A-485 for 6 days. Red arrows indicate patches of TSC-like morphology. Scale bars, 100µm; (B) CREBBP protein levels (~300 kDa) in CREBBP homozygous deletion (clone 1) and CREBBP heterozygous deletion (clone 5) cells determined by Western blot. Lamin B1 (~66 kDa) was used as a loading control. Percentages indicate CREBBP band intensities normalized to Lamin B1 and shown relative to the corresponding mock control.

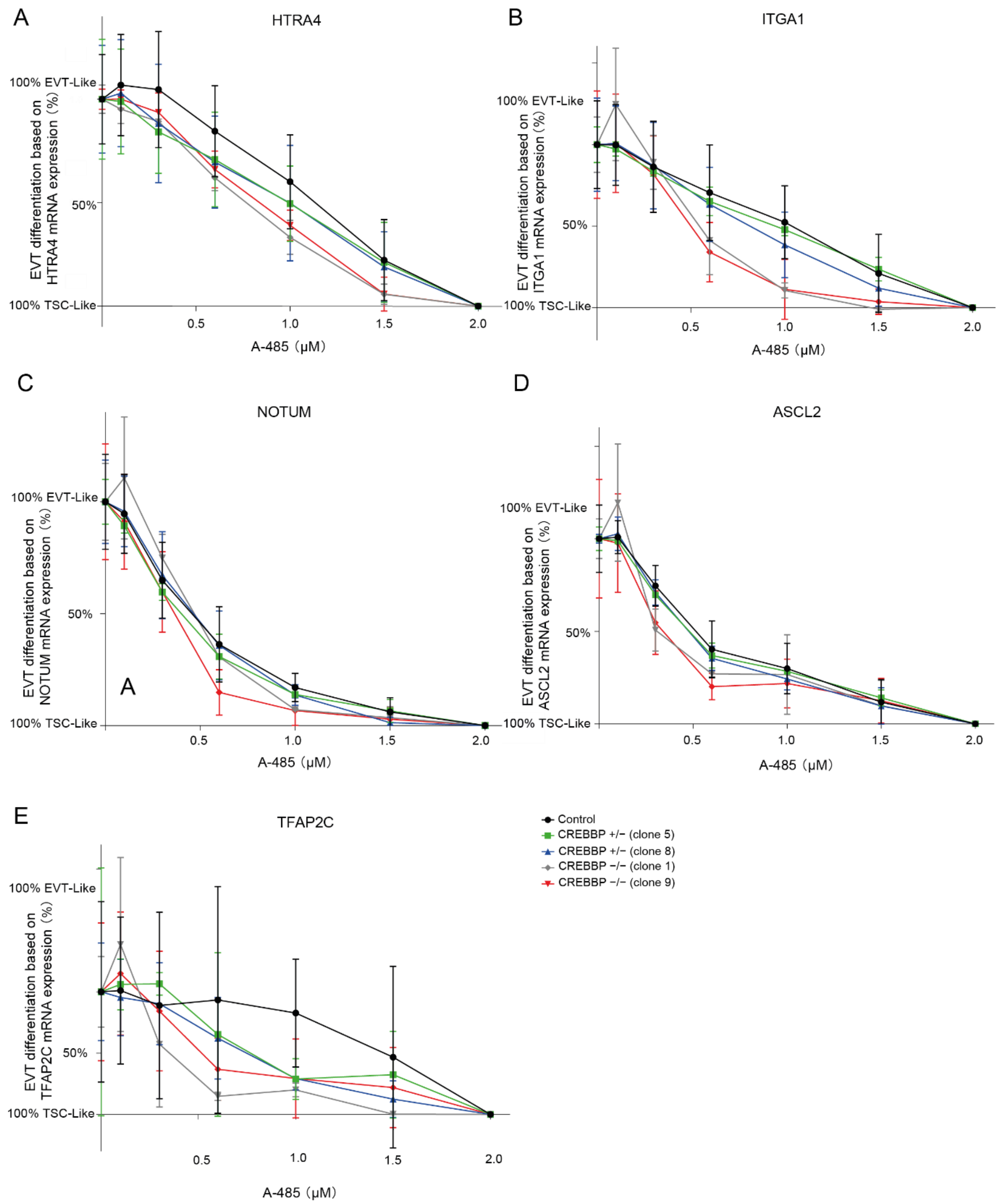

Figure S3. (related to Figure 3). A-485 dose-response analysis of selected trophoblast differentiation markers in CREBBP mutant TSCs. Normalized mRNA expression of (A) *HTRA4*, (B) *ITGA1*, (C) *NOTUM*, (D) *ASCL2*, (E) *TFAP2C* in TSCs control, *CREBBP* +/-, and *CREBBP* -/- mutant cells treated with increasing concentrations of A-485 under EVT differentiation conditions. Expression is shown as a percentage relative to 100% EVT-like and 100% TSC-like reference values. Data represent mean ± SD; n = 3 independent experiments. Two-way ANOVA was used to assess the effects of genotype, A-485 dose, and their interaction. Detailed p values are provided in Table S2.

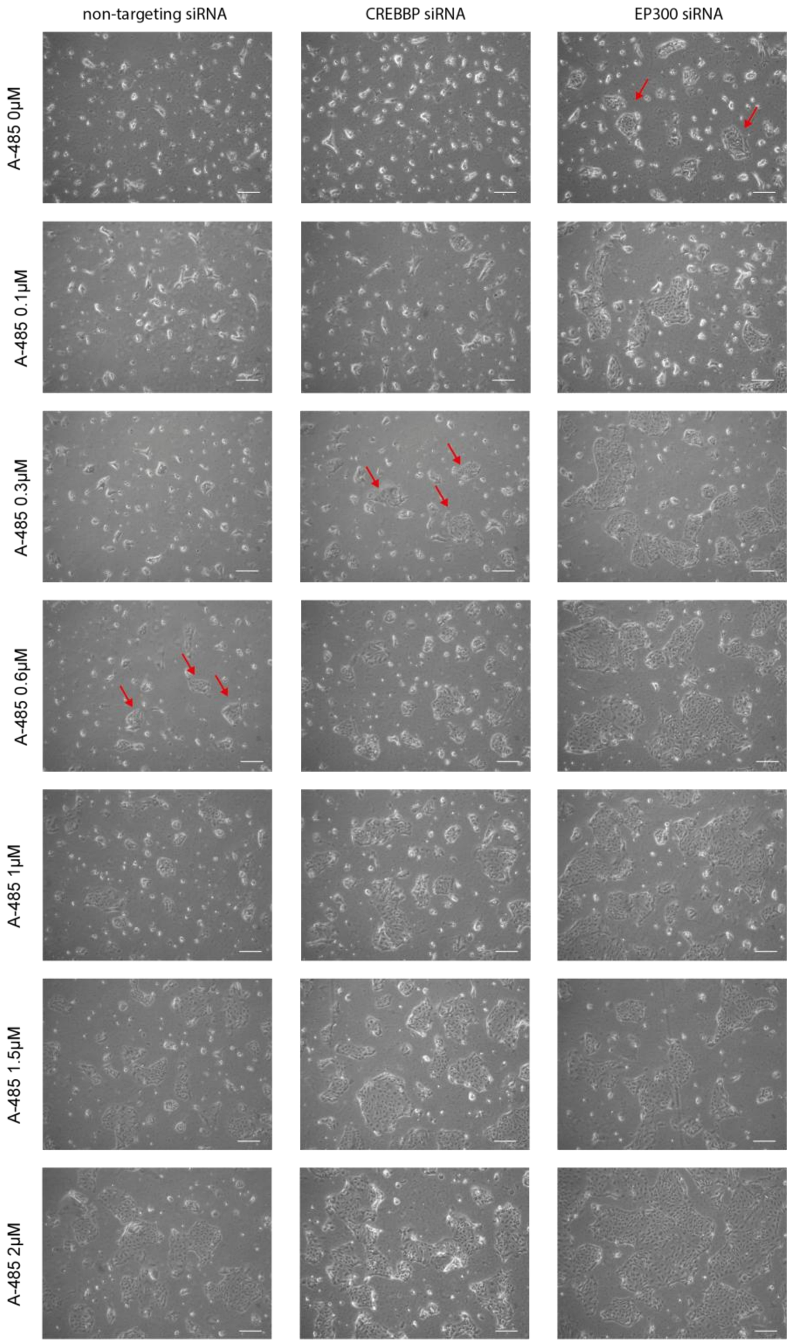

Figure S4. (related to Figure 3). *CREBBP* knockdown increases TSC sensitivity to A-485 during EVT differentiation, but less so than EP300 knockdown. Phase-contrast microscopy images of TSCs transfected with non-targeting siRNA, *CREBBP* siRNA, or *EP300* siRNA and cultured in EVT medium supplemented with increasing concentrations of A-485 for 4 days. Red arrows indicate patches of TSC-like morphology. Scale bars, 250 μm.

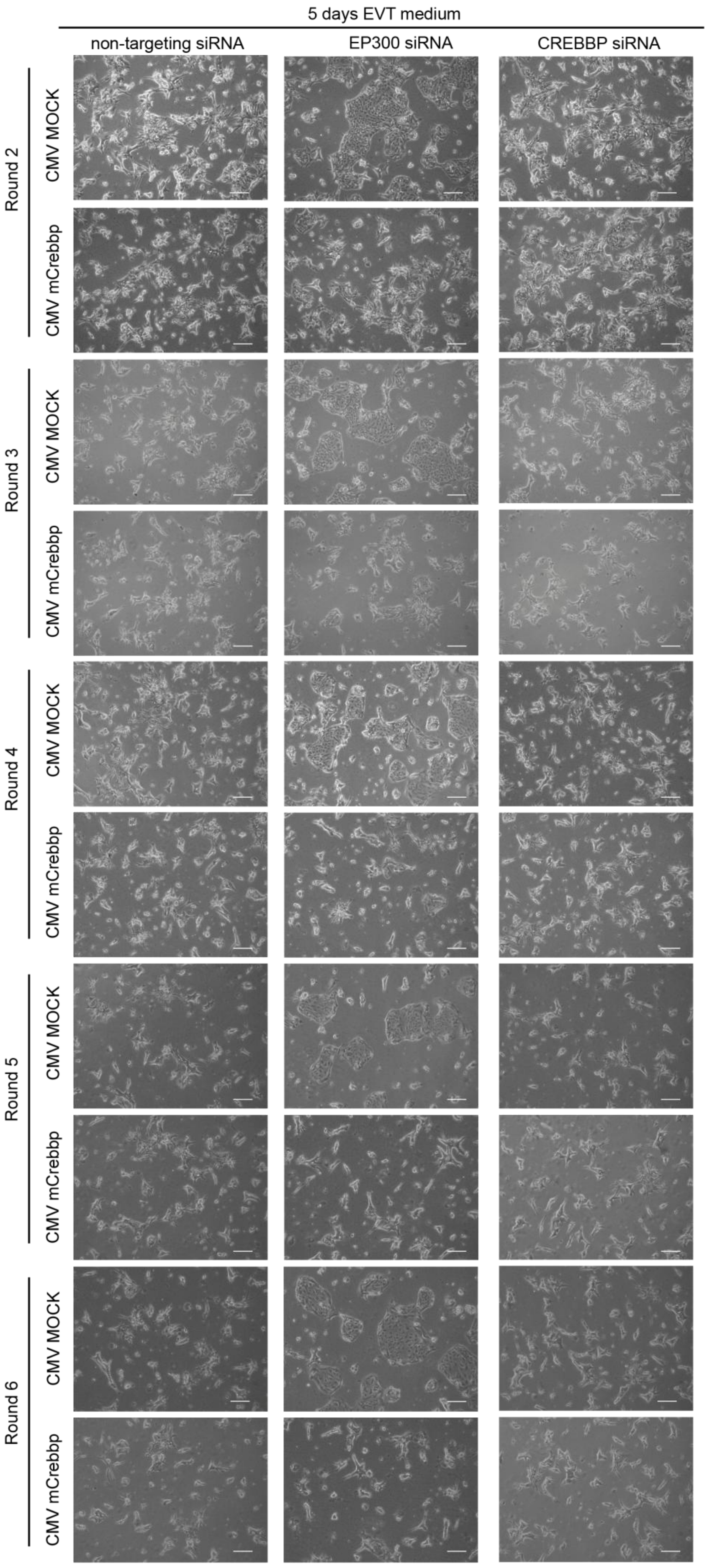
5 days EVT medium
non-targeting siRNA
EP300 siRNA
CREBBP siRNA
Round 2
Round 3
Round 4
Round 5
Round 6
CMV MOCK
CMV mCrebbp

Figure S5. (related to Figure 5). Additional rounds of *mCrebbp* overexpression rescue experiment. Phase-contrast microscopy images of TSCs transduced with CMV mock or CMV *mCrebbp* constructs and subsequently transfected with non-targeting siRNA, *EP300* siRNA, or *CREBBP* siRNA, cultured in EVT medium for 5 days. Images represent independent experimental rounds 2–6. Scale bars, 250 μm.

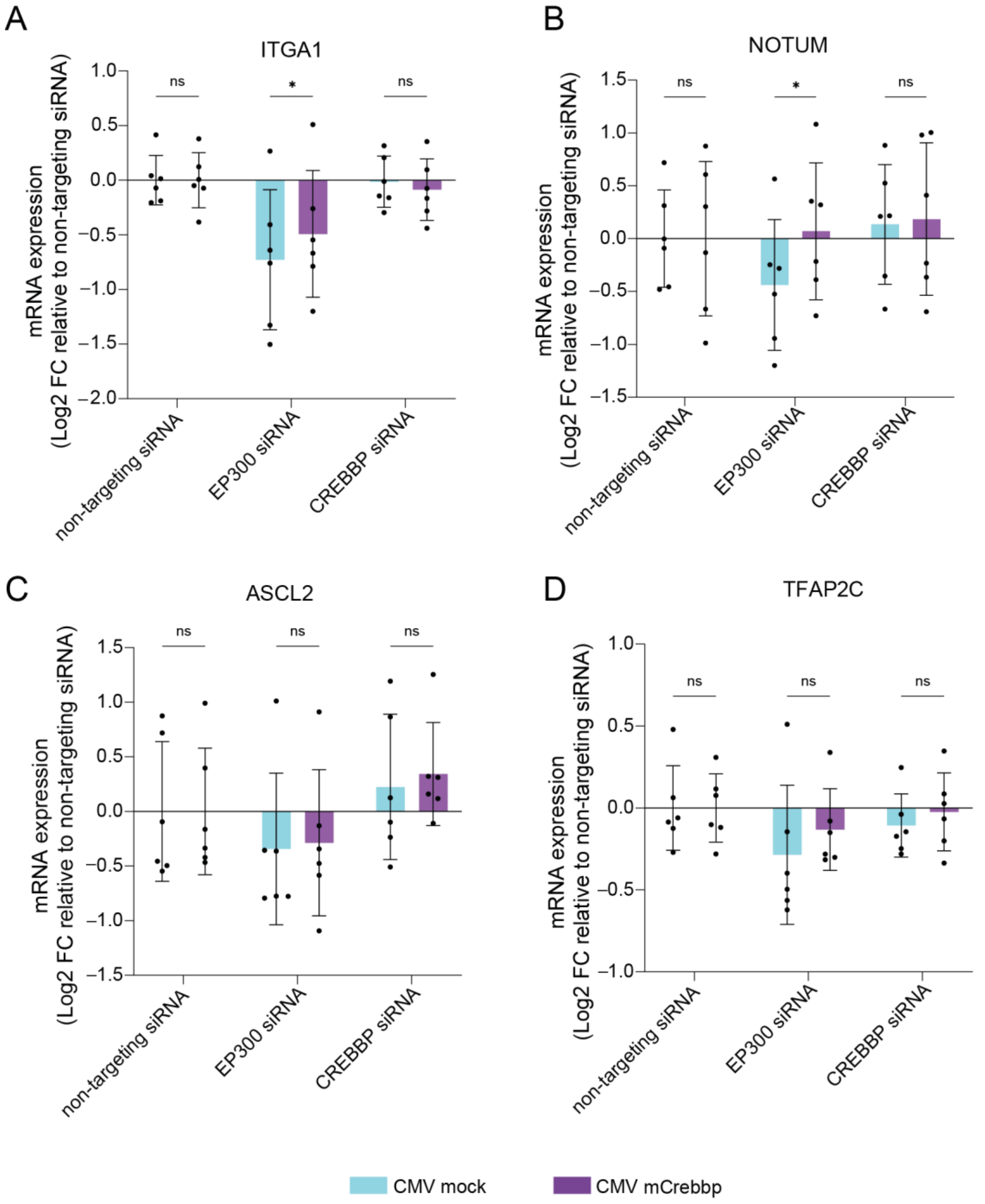

Figure S6. (related to Figure5). Additional qPCR analysis of trophoblast marker expression in the mCrebbp overexpression conditions. mRNA expression of (A) *ITGA1*, (B) *NOTUM*, (C) *ASCL2* and (D) *TFAP2C* in TSCs transfected with non-targeting siRNA, *EP300* siRNA or *CREBBP* siRNA, followed by CMV mock or CMV *mCrebbp* transduction and culture under EVT differentiation conditions for 5 days. Data are presented as mean log2 fold-change ± SD relative to the non-targeting siRNA condition. n = 6 independent experiments. **p* < 0.05; ns, not significant.

Table S1. Genomic characteristics of CRISPR/Cas9-generated CREBBP mutant TSCs.

Name	DNA	Protein
CREBBP +/- clone 5	NG_009873.1:g.[34772_34774delinsG];[=]	NP_004371.2:p.[Gln249ArgfsTer31];[=]
CREBBP -/- clone 1	NG_009873.1:g.[34772_34774];[34774_34775insA]	NP_004371.2:p.[Met250AsnfsTer31];[Gln249HisfsTer31]
CREBBP +/- clone 8	NG_009873.1:g.[34775_34778delinsATG];[=]	NP_004371.2:p.[Met250AsnfsTer7];[=]
CREBBP -/- clone 9	NG_009873.1:g.[34772_34774delinsT];[34773_34774del]	NP_004371.2:p.[Gln249LeufsTer32];[Gln249HisfsTer32]

Table S2. Two-way ANOVA results for A-485 dose-response curves

	A-485 effect	Genotype effect	Interaction
**HLA-G**	****	***	*
**MMP2**	****	***	*
**HTRA4**	****	ns	ns
**ITGA1**	****	**	**
**NOTUM**	****	****	***
**ASCL2**	****	**ns**	**ns**
**TFAP2C**	**ns**	**ns**	**ns**

*$p < 0.05$, **$p < 0.01$, ***$p < 0.001$, ****$p < 0.0001$; ns, not significant .

Table S3. Chemicals used in cell culture

Reagent	Manufacturer	CAT.no.
Collagen IV, Mouse	Corning	354233
DMEM/F-12 + GlutaMAX	Gibco	31331-028
Valproic Acid	STEMCELL Technologies	72292
ITS-X Supplement	Thermo Fisher	51500056
Knockout Serum Replacement	Gibco	10828-010
Bovine serum albumin solution	Merck	A9205
Penicillin-Streptomycin	Gibco	15140-122
L-ascorbic acid	Sigma	A4544
A83-01	STEMCELL Technologies	72022
Y-27632	STEMCELL Technologies	72304
Human Recombinant EGF	STEMCELL Technologies	78136
CHIR99021	STEMCELL Technologies	72052
DMEM	Gibco	41966-052
Fetal Bovine Serum, charcoal stripped	Gibco	12676-011
L-Glutamine GMP	BioWhittaker	BE17-605E
MEM non-essential amino acids	Gibco	11140035
2-Mercaptoethanol	Gibco	31350010
Human Recombinant Heregulin-beta 1	STEMCELL Technologies	78071.1
growth factor-reduced (GFR) Matrigel	Corning	356231
TrypLE Express Enzyme	Thermo Fisher	12604021
0.25% Trypsin-EDTA	Gibco	25200-056

Table S4. siRNA sequences used for RNA interference

siRNA	Manufacturer	Cat.no.	sequence
EP300	QIAGEN	SI02622592	CACCGATAACTCAGACTTGAA
CREBBP	QIAGEN	SI02633085	AACGGAGGTCGCGTTTACATA
Nontargeting	QIAGEN	1027281	Proprietary sequence

Table S5. Primers used for quantitative real-time PCR

Target gene symbol	Forward primer	Reverse primer
TEAD4	CAGGTGGTGGAGAAAGTTGAGA	GTGCTTGAGCTTGTGGATGAAG
MMP2	TGGCACCCATTTACACCTACAC	ATGTCAGGAGAGGCCCCATAGA
HLA-G	CCACCACCCTGTCTTTGACTAT	ACGTCCTGGGTCTGGTCCT
HTRA4	AGGAACCAGCAGTGGATTGAGG	TCCAGCCCGAAGGTCAGATGAT
ITGA1	CCGAAGAGGTACTTGTTGCAGC	GGCTTCCGTGAATGCCTCCTTT
NOTUM	CTACTGGTGGAACGCAAACATGG	CGCACCACCTCCTGGATGATG
ASCL2	CGCCTACTCGTCGGACGACAG	GCCGCTCGCTCGGCTTCCG
TFAP2C	CACCTGTTGCTGCACGATCAGA	AGGAGCGACAATCTTCCAGGGA
EP300	TCTTCAGCACCATGGACAGT	GTTGCATACGAGGCCCATAG
CREBBP	AGTAACGGCACAGCCTCTCAGT	CCTGTCGATACAGTGCTTCTAGG
mCrebbp	CACCATCTGTGGCTACTCCTCA	GGTTTCAGCACTGGTCACAGAG
EEF2	CTGGAGATCTGCCTGAAGGA	CGACCGGGTCAGATTTCTT
GUSB	GGAGTGCAAGGAGCTGGAC	ATTGAAGCTGGAGGGAACTG
PSMD4	CTGGCTAAACGCCTCAAGAAGG	CACTGTCACCAGATGAGAACCG

Table S6. Antibodies used for Western blotting.

Target Protein	Manufacturer	Cat. No.	Source	Clone	Dilution
CREBBP	Cell Signaling Technology	7389	Rabbit	D6C5	1:1000
Lamin B1	Abcam	AB133741	Rabbit	EPR8985(B)	1:1000
